# Satellitome analysis on the pale-breasted thrush *Turdus leucomelas* (Passeriformes; Turdidae) uncovers the putative co-evolution of sex chromosomes and satellite DNAs

**DOI:** 10.1038/s41598-024-71635-5

**Published:** 2024-09-04

**Authors:** Guilherme Mota Souza, Rafael Kretschmer, Gustavo Akira Toma, Alan Moura de Oliveira, Geize Aparecida Deon, Princia Grejo Setti, Rodrigo Zeni dos Santos, Caio Augusto Gomes Goes, Analía Del Valle Garnero, Ricardo José Gunski, Edivaldo Herculano Correa de Oliveira, Fabio Porto-Foresti, Thomas Liehr, Ricardo Utsunomia, Marcelo de Bello Cioffi

**Affiliations:** 1 Departamento de Genética e Evolução, Universidade Federal de São Carlos, São Carlos, SP 13565-905 Brazil; 2 Departamento de Ecologia, Zoologia e Genética, Instituto de Biologia, Universidade Federal de Pelotas, Pelotas, RS 96010-610 Brazil; 3 Faculdade de Ciências, Universidade Estadual Paulista, Bauru, SP 17033-360 Brazil; 4 Universidade Federal do Pampa, Campus São Gabriel, São Gabriel, RS 97307-020 Brazil; 5 Seção de Meio Ambiente, Instituto Evandro Chagas, Ananindeua, PA 67030-000 Brazil; 6 Instituto de Ciências Exatas e Naturais, Universidade Federal do Pará, Belém, PA 66075-110 Brazil; 7 Institut für Humangenetik, Universitätsklinikum Jena, Friedrich-Schiller Universität, 07747 Jena, Germany

**Keywords:** Molecular cytogenetics, Evolution, Neo sex chromosomes, Translocation, satDNA, Cytogenetics, Evolutionary biology, Genomics

## Abstract

Do all birds' sex chromosomes follow the same canonical one-way direction of evolution? We combined cytogenetic and genomic approaches to analyze the process of the W chromosomal differentiation in two selected Passeriform species, named the Pale-breasted Thrush *Turdus leucomelas* and the Rufous-bellied thrush *T. rufiventris*. We characterized the full catalog of satellite DNAs (satellitome) of *T. leucomelas*, and the 10 TleSatDNA classes obtained together with 16 microsatellite motifs were in situ mapped in both species. Additionally, using Comparative Genomic Hybridization (CGH) assays, we investigated their intragenomic variations. The W chromosomes of both species did not accumulate higher amounts of both heterochromatin and repetitive sequences. However, while *T. leucomelas* showed a heterochromatin-poor W chromosome with a very complex evolutionary history, *T. rufiventris* showed a small and partially heterochromatic W chromosome that represents a differentiated version of its original autosomal complement (Z chromosome). The combined approach of CGH and sequential satDNA mapping suggest the occurrence of a former W-autosomal translocation event in *T. leucomelas*, which had an impact on the W chromosome in terms of sequence gains and losses. At the same time, an autosome, which is present in both males and females in a polymorphic state, lost sequences and integrated previously W-specific ones. This putative W-autosomal translocation, however, did not result in the emergence of a multiple-sex chromosome system. Instead, the generation of a neo-W chromosome suggests an unexpected evolutionary trajectory that deviates from the standard canonical model of sex chromosome evolution.

## Introduction

Nearly all bird species share the same ZZ/ZW sex chromosome system (an exception to this rule is described by Ref.^1^, which is widely regarded as a stable sex system with males (ZZ) and females (ZW) representing the homogametic and heterogametic sex, respectively^2,3^. Both the Z and W sex chromosomes originated from an ancestral autosomal pair more than 110 Mya ago^4,5^. In most species (except the Ratites where the sex chromosomes are homomorphic), the W chromosome is small and mostly heterochromatic, whereas the Z is typically preserved in both morphology and gene content^3,6–10^. Despite the conservation of the Z chromosomes, they are subject to frequent intrachromosomal rearrangements, such as inversions, resulting in changes in their morphology within species^2^.

With over 6500 species, the order Passeriformes is the most varied group of birds^10^. Despite this remarkable variety, only ~ 460 of these species, or ~7.0% of the total in this order, have had their diploid number determined^11^. Most Passeriform species have small W chromosomes that have experienced dynamic processes of constitutive heterochromatin accumulation and sequence elimination throughout their evolutionary history^12–14^. Among them, with 88 species, the genus *Turdus* (Thrushes) stands out as the most diverse one^10^. While all Thrushes share common morphological characteristics, they exhibit a wide range of plumage colorations and ecological adaptations. This plasticity allows them to thrive in diverse biomes, including savannahs, alpine areas, and both tropical and temperate forests^15^.

Among the 88 species within the *Turdus* genus, 18 of them have had their karyotypes described, revealing a substantial degree of chromosome similarity among them. The diploid number (2n) ranges from 78 to 84, indicating slight variations^11^. Molecular cytogenetics studies using chicken macrochromosome probes (GGA1-10) have been conducted on a limited scale, encompassing only four species: *T*. *merula*, *T*. *iliacus*, *T*. *rufiventris*, and *T*. *albicollis*^16–18^. These investigations unveiled a sole interchromosomal rearrangement, specifically the fission of the ancestral chromosome one (GGA1), which is a common characteristic observed among Passeriforms^2,11^. In *T. merula,* except for chromosome 16, which has remained unstudied, no evidence of interchromosomal rearrangements in connection to the homologous chromosomes to GGA11-28 has been found in any of the microchromosomes^19^.

In recent years, the integration of molecular cytogenetics techniques with in silico data derived from the Next Generation Sequence (NGS) and novel software pipelines have provided significant advances in the comprehension of intricate chromosome rearrangements^20–22^ and in the evolution of sex chromosomes^23,24^. In particular, the characterization and the in situ mapping of the satelitome, which is a catalog of the most representative satellite DNAs (satDNAs) in a genome^25^, is capable of highlighting transpositions and translocation events, giving insights into the framework of karyotype evolution and chromosome speciation^26–29^. Moreover, although still incipient in birds (however see^30,31^), investigations in other vertebrates, such as mammals^32–37^ and amphibians^38,39^, demonstrated the fast-evolving nature of these *in tandem* repetitive DNAs and their putative role in the formation and composition of centromeres and in the evolution of sex chromosomes^35,40,41^.

Comparative genomic hybridization (CGH) (sometimes also referred as GISH) is a fine-scale molecular cytogenetic approach used to detect chromosomal rearrangements that has also been applied to discover the evolutionary origin and composition of sex chromosome systems^42,43^. This method allows us to recognize the high level of molecular differentiation of sex chromosomes, localize sex-specific chromosome regions, and to track early stages of sex chromosome differentiation in several groups^44,45^. In this way, repetitive DNAs and comparative genomic hybridization mapping, are an attempt to advance toward the knowledge of the processes that have shaped the evolution of sex chromosomes.

Here, we selected two Passeriform species belonging to the Turdidae family, named the Pale-breasted thrush *T. leucomelas* and the Rufous-bellied thrush *T. rufiventris* to analyze the process of their W chromosomal evolution. In that regard, we compared the intragenomic differences (focusing on their repetitive DNA content) between males and females of each species and used cytogenetic and genomic methods to analyze their satDNA composition and their putative involvement in their W chromosomal evolution.

## Results

The aim of our work was to characterize and map the satellite DNA sequences present in the species *T. leucomelas* (TLE) and then compare these sequences isolated in a similar species, *T. rufiventris* (TRU). First, we investigated and confirmed that the 2n for both species investigated were 2n = 80 for *T. leucomelas* and 2n = 78 for *T. rufiventris.* These results corroborated earlier information for these species^18,46^. The next step in delving deeper into the previously mentioned issues was to describe *T. leucomelas*´ satellitome.

### satDNA content of the *T. leucomelas*´ genome

After three iterations in TAREAN, 10 satDNA families (TleSatDNAs) were recovered. Table 1 presents the general characteristics of the *T. leucomelas´* satellitome, such as the A + T content of the satellites, which ranged from 27.2 to 69.6%, with an average of 51.73%, and the length of the repeated units (RUL), which ranged between 21 and 1644 bp with 80% of the satDNAs families having monomers greater than 100 bp. By aligning each *T. leucomelas* satDNA in the RM_Homology version 1 (https://github.com/fjruizruano/satminer) and Geneious software version 8.0 (https://www.geneious.com), a superfamily relationship (50–80% similarity) was observed between the satDNAs TleSat02-145 and TleSat05-21, which are considered a *high-order repeat* (HOR). The *repeat landscapes* generated are shown in Supplementary Fig. S1. Comparing the satDNA catalogs of males and females, the presence of two satellites more abundant in females than in males was observed, these being TleSat06-645 (ratio of 3.94 between genders) and TleSat08-419 (ratio of 51.89 between genders) (Table 1).

**Table 1:** General features of *T. leucomelas* satellitome.

satDNA	RUL	Abundance (F)	Abundance (M)	Abundance (F/M)	A + T (%)
TleSat01-1220	1220	0.043067479	0.052959842	0.813210121	48
TleSat02-145	145	0.000925024	0.000867871	1.065853631	46.9
TleSat03-1644	1644	0.000871392	0.00093426	0.932706941	49.8
TeleSat 04-23	23	0.000508905	0.00088081	0.577765438	69.6
TleSat05-21	21	0.00048423	0.00047336	1.022953453	47.6
TleSat06-645	645	0.00046497	0.00011781	3.94678455	62.8
TleSat07-103	103	0.00033896	0.00048223	0.702890814	27.2
TleSat08-419	419	0.000211289	4.07E-06	51.89737354	60.4
TleSat09-638	638	0.000164396	0.00015275	1.076197143	41.2
TleSat10-426	426	0.000140907	0.00025902	0.543988135	63.8

*RUL* repeat unit length, *F* female, *M* male, *A* + *T* adenine and thymine content.

**Table 2:** List of analyzed species, with the indication of the respective collection location, sample number (N), sex of individuals collected, and the code applied for all individuals analyzed.

Species	Location	N	Individuals
*Turdus leucomelas* (TLE)	Porto Vera Cruz (RS), Brazil	(02♀; 03♂)	F01, F02; M01, M03, M04
*Turdus leucomelas* (TLE)	Belém (PA), Brazil	(–♀; 01♂)	M02
*Turdus rufiventris* (TRU)	São Gabriel (RS), Brazil	(02♀; 02♂)	F01, F02, M01, M02

*RS* Rio Grande do Sul, *PA* Pará (Brazilian States).

### Minimmum spanning trees (MSTs)

**Fig. 1:**
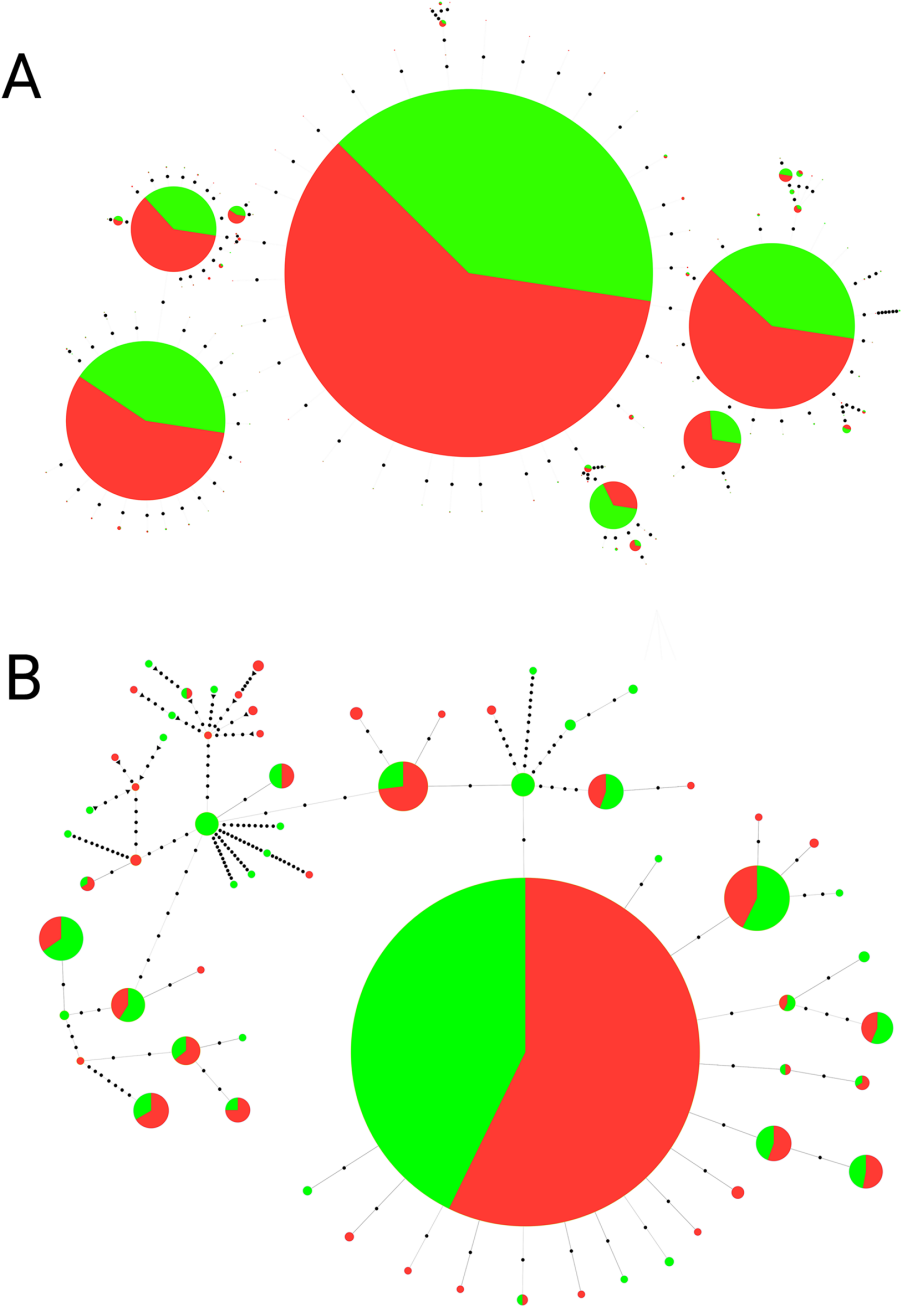
Linear MSTs of (**A**) TleSat05-21 and (**B**) TleSat07-103 obtained from female (red) and male (green) reads. Each circle represents one haplotype and the diameter is proportional to the abundance of the haplotype. Black dots represent a mutation event.

**Fig. 2:**
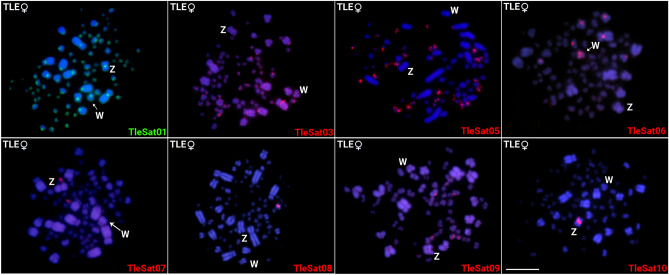
Chromosomal mapping of the eight TleSatDNAs hybridized on female metaphases of *T. leucomelas* (TLE ♀F01). The Z and W sex chromosomes are indicated. While the Z chromosome was identified by its distinct morphology (i.e., the only metacentric macrochromosome), the W chromosome was appropriately identified after a sequential hybridization with TleSat06, which provides a unique and distinctive pattern for this chromosome. Bar 10 μm.

We selected TleSat05-21 and TleSat07-103 to generate minimum spanning trees (MSTs) (Fig. 1). These satDNAs were selected due to their monomer sizes (< 150 bp), differential abundance between sexes (see Table 1), and clusterization after FISH results (Fig. 2). TleSat05-21 doesn’t demonstrate accumulation in the sex chromosomes of *T. leucomelas* (Fig. 2), and the MST is composed of six mainly haplotypes shared between males and females, following the observed ratio of abundance in males and females (1.02, Table 2). In contrast, TleSat07-103 shows a ratio of 0.70, with more abundance of this sequence in males than in females. The MST demonstrates a predominance of one haplotype, shared between sexes, and several less abundant haplotypes, and some of them are male-specific (Fig. 1), despite the absence of FISH signals in the Z chromosome.

### Chromosomal distribution of TleSatDNAs and microsatellites

Following the in situ investigations, we found that*,* Except for TleSat02 and TleSat04, all the remaining TleSatDNAs showed positive signals on female chromosome metaphases of *T. leucomelas* (Fig. 2). The TleSat01 displayed signals in the centromeric region of all chromosomes. TleSat03 was mapped in the centromeric region of two pairs of macrochromosomes, as well as in some microchromosomes. TleSat06 was located in the pericentromeric region of three macrochromosomes, one microchromosome, and on the W. TleSat05, TleSat07, and TleSat09 were exclusively mapped on microchromosomes, while TleSat10 was exclusively mapped on the pericentromeric region of the Z chromosomes (Fig. 2). The TleSat06 and TleSat08 displayed a variable number of sites among the individuals, indicating a polymorphism related to the satellites, which may involve W-autosomal translocation events (as will be further discussed) or also to transpositions of mobile elements.

**Fig. 3:**
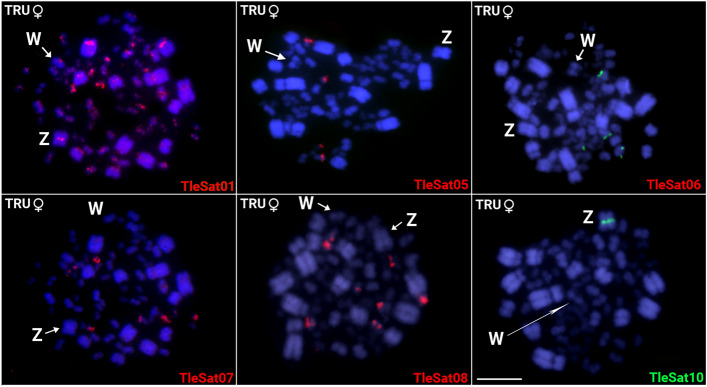
Chromosomal mapping of TleSatDNAs on metaphase plates of T. *rufiventris* (TRU ♀F01). While the Z chromosome was identified by its distinct morphology (i.e.: the only metacentric macrochromosome), the W chromosome was appropriately identified after a sequential C-banding, which provides a unique and distinctive pattern for this chromosome. The Z and W sex chromosomes are indicated. Bar 10 μm.

In *T. rufiventris,* only six of the 10 TleSatDNA (TleSat01, TleSat05, TleSat06, TleSat07, TleSat08, and TleSat10) showed positive signals after in situ experiments (Fig. 3). The TleSat01 and TleSat10 present the same pattern found in *T. leucomelas*, being mapped in all centromeres and solely on the Z chromosome, respectively. However, TleSat05, TleSat06, TleSat07, and TleSat08 showed different accumulations in *T. rufiventris.* Although TleSat05 and TleSat07 exhibited hybridization signals only in the microchromosomes, like in *T. leucomelas*, no signals for TleSat06 were observed in the W chromosome of this species. TleSat08 only displayed hybridization clusters in a few pairs of microchromosomes (Fig. 3).

**Fig. 4:**
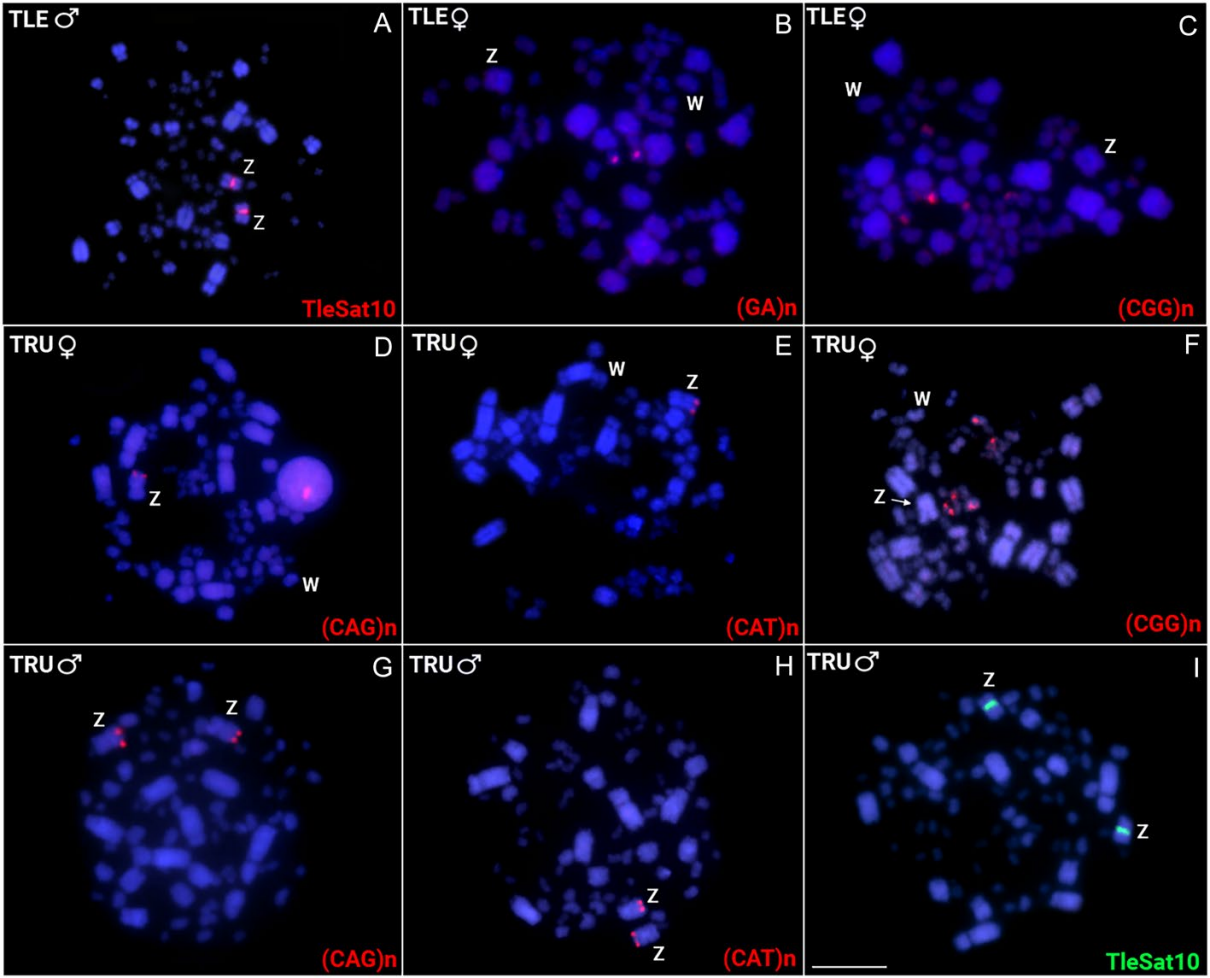
Metaphase plates of *T. leucomelas* TLE♂M01; TLE♀F01; *T. rufiventris* TRU♀F01 and TRU♂M01 highlighting the chromosomal mapping of microsatellites (**B**–**H**) and TleSat10 (**A**,**I**). Bar 10 μm.

Of the total of 16 microsatellites tested in both Thrushes, only two had positive hybridization signals in *T. leucomelas,* named (GA)_15_ and (CGG)_10_, which demonstrated clusters in one and three pairs of microchromosomes, respectively (Fig. 4). On the other hand, *T. rufiventris* showed positive hybridization signals for three microsatellites, with (CGG)_10_ displaying signals in three pairs of microchromosomes, while (CAG)_10_ and (CAT)_10_ both accumulated in the telomeric region of Z chromosomes (Fig. 4).

### Comparative genomic hybridization

**Fig. 5:**
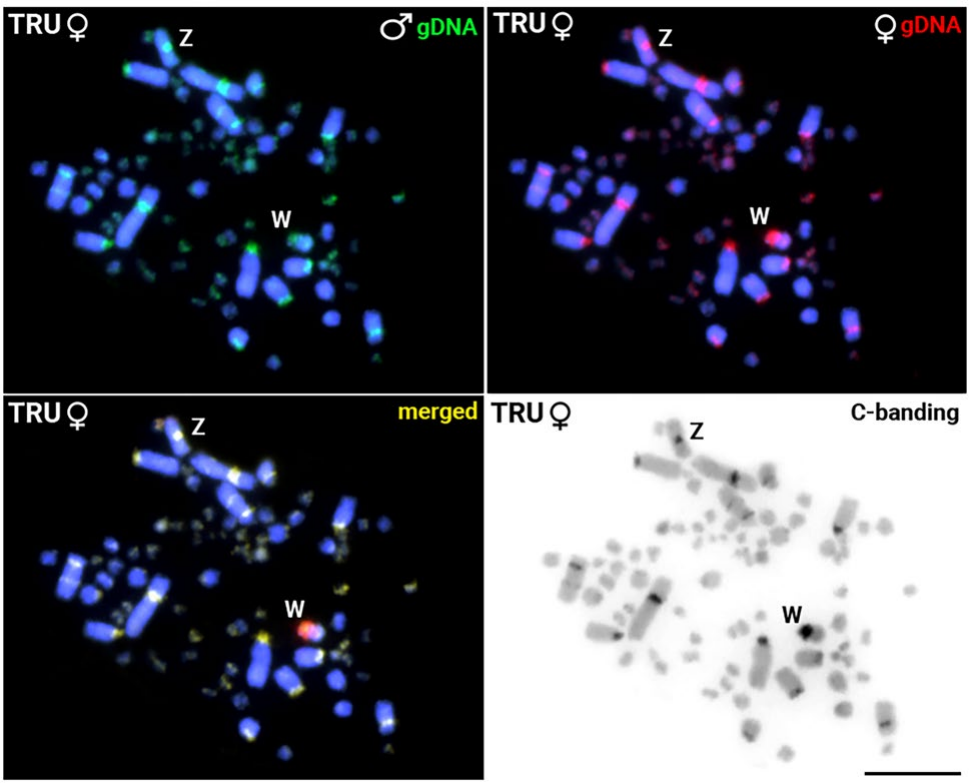
*Turdus rufiventris* male and female genomic DNA probes hybridized on female metaphase chromosomes of *T. rufiventris* (TRU♀F01) following the experimental design described in Table 3. The hybridization patterns of the probes derived from male (green), female (red), and the combined pictures are shown in (**A**), (**B**), and (**C**), respectively. The sequential C-banding highlighted a conspicuous C-positive heterochromatic block in the short arms of the W chromosome (**D**). Bar 10 μm.

**Fig. 6:**
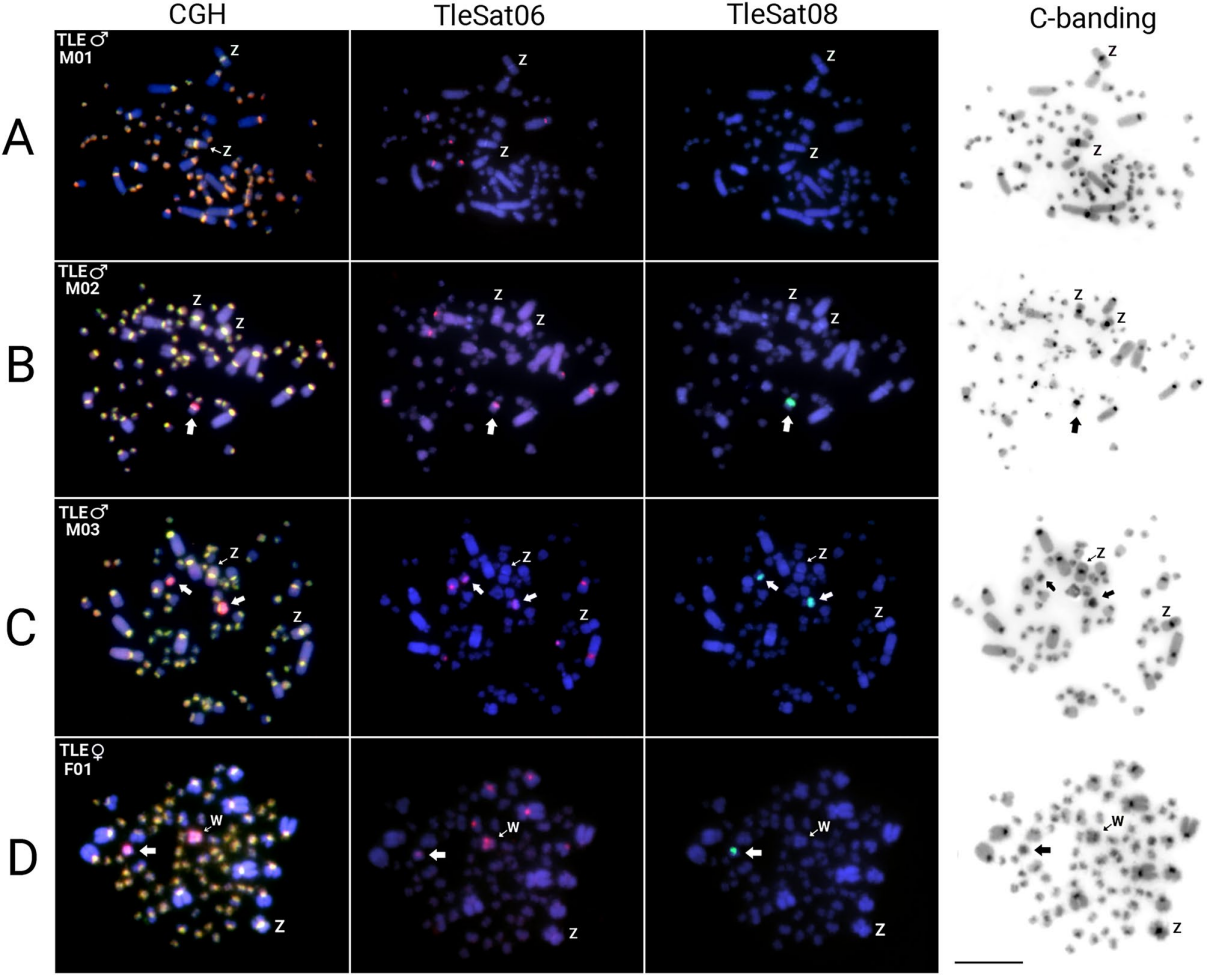
Intraspecific genomic hybridization (CGH) in *T. leucomelas* males: (**A**) TLE♂M01; (**B**) TLE♂M02; (**C**) TLE♂M03 and female (**D**) TLE♀F01 specimens following the experimental design described in Table 3. The merged images displayed in the CGH column were obtained from those present in Supplementary Figs. S2 and S3. After the CGH, chromosomes were sequentially mapped with TleSat06 (second column) and TleSat08 (third column) probes, and then C-banded (fourth column). The small autosomes displaying female-biased hybridization signals, the accumulation of Tlesat06 and TleSat08, and a conspicuous C-positive block are indicated by the arrowheads. Bar 10 μm.

Lastly, after examining specific sequences for each sex, we found overlapping signals in the pericentromeric regions of almost all chromosomes, except for an exclusive strong female-specific region on the W chromosome, coincident with a C-positive heterochromatic block (Fig. 5, Supplementary Fig. S2E). Contrarily, four distinct hybridization patterns were identified in the *T. leucomelas* individuals (Fig. 6, Supplementary Figs. S2 and S3). In addition to overlapping signals in the centromeric region of all male and female chromosomes, the accumulation of female-biased hybridization signals in the entire W chromosome and half of a small autosome was evidenced in the two females (TLE♀F01 and TLE♀F02) analyzed (Fig. 6D, Supplementary Fig. S3). In turn, three different hybridization patterns were found in the four males analyzed. In the TLE♂M01 only overlapping signals of the male and female gDNA probes were detected in the centromeric region of all chromosomes (Fig. 6A, Supplementary Fig. S2A). This same pattern was also observed in the other three males, in addition to one copy (in TLE♂M02) or two copies (in both TLE♂M03 and TLE♂M04) of the same small autosome displaying female-biased hybridization signals (Fig. 6B,C**,** Supplementary Fig. S2B–D). We sampled individuals from different populations, thus ensuring that the patterns discovered were not exclusive to a specific population. In all cases, this small autosome also accumulated the TleSat06 and TleSat08 (Fig. 6). While the whole short arms of the W chromosome contain a weak C-positive heterochromatic block, prominent C-positive blocks are observed in the Z chromosome and in the short arms of the small autosome that exhibit the female-biased hybridization signals (Fig. 6).

## Discussion

The diploid numbers of both species, *T. rufiventris* and *T. leucomelas*, have already been characterized in previous works, like the morphology of their ZW sex chromosomes^18,46^. However, despite their similar sizes, no data on the molecular and heterochromatic content of their W chromosomes was currently available. Here, we provide further confirmation of the 2n number [i.e.: *T. leucomelas* (2n = 80) and *T. rufiventris* (2n = 78)] and molecular cytogenetic analyses. We showed that the W chromosome of *T. rufiventris* has a very strong C-positive band on its short arms (Fig. 5), while the W chromosome of *T. leucomelas* exhibited a faint block of heterochromatin encompassing just its entire short arms (Fig. 6). Although the occurrence of W chromosomes with unusual morphologies and scarce in heterochromatin has also been evidenced^47,48^, both these patterns seem atypical for W chromosomes of Passeriformes members, once most species up to now karyotyped have almost entirely heterochromatic W chromosomes, such as in the zebra finch (*Taeniopygia guttata*), the canary (*Serinus canaria*)^14^, and the Sooty-fronted Spinetail (*Synallaxis frontalis*)^49^.

To characterize the repetitive DNA fraction of these W chromosomes, we first isolated and characterized the satellitome of *T. leucomelas* and further in situ mapped the 10 TleSatDNAs obtained, together with 16 microsatellite motifs in both *T. leucomelas* and *T. rufiventris* species. The data confirm the previous findings obtained in some few bird species highlighting that avian satellites are usually composed of a small number of particularly large satDNAs rich in GC content^30^. However, this is the first case where the satellitomes were mapped in their respective chromosomes. The MSTs produced in this work demonstrate a prevalence of shared haplotypes between males and females to TleSat05 and TleSat07, due to their presence in autosomal microchromosomes, as demonstrated by FISH. The presence of TleSat07 haplotypes exclusive to males, together with its higher frequency in males, implies the presence of Z chromosome clusters that are not visible by FISH, possibly because of the small array sizes (Figs. 1 and 2).

Except for TleSat01, which is present in the centromeric region of all *T. leucomelas* and *T. rufiventris* chromosomes (probably representing their primary centromere component), and TleSat06, which accumulated exclusively in *T. leucomelas*' W chromosome, we did not detect any evidence of accumulation for the extant TleSatDNAs on the W chromosomes of both species (Figs. 2 and 3). Similarly, none of the microsatellites examined in this study were accumulated on any W chromosomes (Fig. 4). The heterogametic chromosomes (W and Y) tend to differentiate once recombination ceases and heterochromatization followed by the accumulation of repetitive elements begins^50,51^. In this pathway, Ref.^52^ proposed that the accumulation of satDNA sequences throughout the length of the sex-specific (Y and W) chromosome plays a significant role in generating its morphological differentiation from the X or Z, respectively. Likewise, microsatellite repeats are crucial for the differentiation of sex-specific chromosomes, as they may be the first type of repeat that accumulates during its early stages of differentiation^53,54^. Accordingly, reports from various taxa show the accumulation of repetitive sequences specifically on the Y or W chromosomes, which are enriched in high-, middle-, and low-copy repetitive sequences and contain only a few functional genes^35,38,41,51,55,56^. However, it is not a rule that most repetitive sequences are found exclusively in heterogametic chromosomes, as revealed by several groups^57–59^. Here, an exceptionally high number of repetitions accumulated on the Z chromosomes, including centromeric clusters of TleSat10 in both species as well as (CAG)n and (CAT)n in the terminal region of the q arms of the Z chromosomes of *T. rufiventris* (Figs. 2, 3, 4). This scenario is unusual among birds since very few cases of repeat accumulation on the Z chromosomes were documented^59–62^.

Instead, the great majority of TleSatDNAs was mapped in microchromosomes in both species. Experiments in other bird families, including Caprimulgidae and Picidae, have also demonstrated a high density of repetitive microsatellite and telomeric sequences in microchromosomes^59,63,64^. Similarly, in some species of turtles and lizards, the accumulation of these repeats in microchromosomes has also been shown^65,66^.

**Fig. 7:**
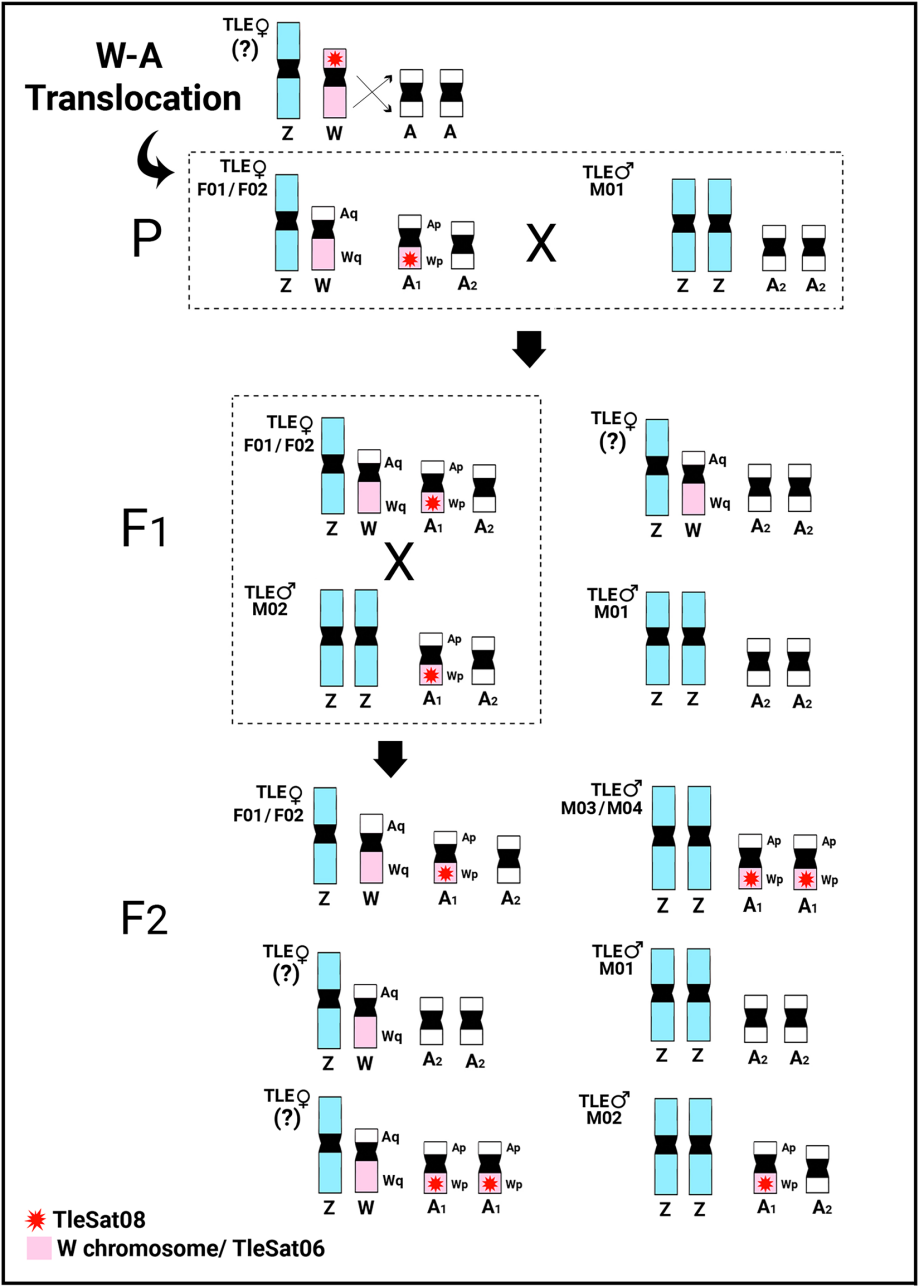
Idiogram representing one of the main hypotheses that involve a translocation event in *T. leucomelas* specimens and the resulting genotypes from possible crossings. Under this hypothesis, an ancestral female undergoes a translocation between the short arms of the W chromosome and an autosome (A). As a result, the W chromosome both acquired and lost sequences, while an autosome (A1) also lost sequences and integrated previously W-specific sequences, which included both TleSat06 and TleSat08, while its homologous remained untouched (A2). This pattern (ZW + A1A2) is observed in both *T. leucomelas* (TLE♀F01/F02). When crossed with a wild-type ZZ + A2A2 male (TLE♂M01), it produces an F1 offspring with four possible results: (i) ZW + A1A2 females (TLE♀F01/F02); (ii) ZW + A2A2 females (?); (iii) ZZ + A1A2 males (TLE♂M02), and (iv) ZZ + A2A2 males (TLE♂M01). A new crossing between TLE♀F01/F02 (ZW + A1A2) x TLE♂M02 (ZZ + A1A2) generates an F2 offspring with six possible results: (i) ZW + A1A2 females (TLE♀F01/F02); (ii) ZW + A2A2 females (?); (iii) ZW + A1A1 females (?); ZZ + A1A1 males (TLE♂M03/M04), and (iv) wild-type ZZ + A2A2 males (TLE♂M01). Individuals marked with (?) stand for those that we were unable to find in this work.

In both thrush species, the conventional chromosomal analysis, C-banding, and repetitive DNA mapping pointed to a specific W chromosome arrangement that differs from the majority of avian species up to now analyzed^3,14^, since it does not reveal many repeated sequences or significant blocks of heterochromatin accumulating on chromosomes. In addition, this particular scenario was shown to be even more complex when intraspecific CGH analyses were performed. While the *T. rufiventris* specimens presented the expected overall results after intraspecific-CGH experiments (i.e., the W chromosome showing the only particularly rich region in the female-biased hybridization signals), an unusual pattern was observed in the *T. leucomelas* individuals (Fig. 6). In the two females of *T. leucomelas* (TLE♀F01 and TLE♀F02) analyzed, besides the entire W chromosome, half of a small autosome is also enriched by the female-biased hybridization signals. Except for the TLE♂M01 specimen, the other males show a polymorphic state for this same small autosome, i.e., with only one copy (TLE♂M02) or two copies (both TLE♂M03 and TLE♂M04) of those female-biased hybridization signals (Fig. 6, Supplementary Figs. S2 and S3). So, it is likely that this portion of the autosome enriched by the female-biased hybridization signals was originally part of the W chromosome. Therefore, the occurrence of a W-autosomal reciprocal translocation (not involved in the creation of a multiple-sex chromosome system) is one of the hypotheses that best explains this complex scenario, where the W chromosome both gained and lost sequences^67^, as well as the small autosome (also present in males) which incorporated both TleSat06 and TleSat08 (the latter, being previously W-specific and now present in its short arms) (Fig. 7). Likely, this chromosomal rearrangement does not lead to a dosage composition problem for individuals exhibiting either the heteromorphic or homomorphic condition, as the translocated segment encompasses repetitive DNA sequences (as shown by our CGH-SatDNA-FISH analysis), which are usually transcriptionally silenced^67,68^. However, we cannot exclude an alternative hypothesis, as the presence of repetitive sequences may significantly change due to various parameters, such as copy number variation (expansions and contractions), their genome location, and sometimes even as a result of transposition events and/or major chromosomal rearrangements^67–69^.

How do sex chromosomes evolve? Up until recently, it was widely believed that the sex chromosomes followed a canonical one-way direction of evolution, which was proposed by gathering information from multiple independent works^6,70–72^. This standard sex chromosome evolution model predicts that the Y and W chromosomes gradually differentiate and most of their genetic material is lost owing to a lack of recombination with the X or Z, respectively. This leads to the usual gradual loss of genes or gene function and structural modifications like deletions and heterochromatinization. As a result, the sex-specific chromosome might progressively shrink and ultimately be eliminated from the genome^73–75^. At first glance, since all Neognathae species, with a few exceptions, have small and heterochromatic W chromosomes, this seemed to be also the standard path taken by all bird sex chromosomes (reviewed in^76^ and ^77^. Besides, Ratite birds, which represent the basal avian lineage (paleognaths), present sex chromosomes at an early stage of differentiation, where Z and W chromosomes are still morphologically similar^8,78–80^. Our data in *T. leucomelas*, however, points to an unusual evolutionary pathway for the W chromosome that deviates from the standard canonical model of sex chromosome evolution.

Novel investigations (see for example^81–85^ are steadily describing new deviant models that differ from the canonical one-way direction of evolution. In particular, cases of new genetic material being added to the sex chromosomes are outstanding examples as they contradict the so-thought inevitable degeneration of the heteromorphic sex chromosome^75,85^. In these models, new linkage groups can be created by extensive amplification of sequence copy number, brought by molecular drive, and/or sex chromosome-autosome translocations^85^. In the latter, a reciprocal translocation (i.e., DNA segments are swapped mutually between chromosomes), results in two possible scenarios. The first, leads to a multiple-sex chromosome system, as the two linkage groups, being consequently whole chromosomes, remain in the form of the larger translocation product (e.g., Neo-sex chromosome)^86^. The second scenario, however, does not alter the 2n number, and consists of a non-homologous exchange between different sections of two or more chromosomes, thus generating independent Neo-chromosomes that share common DNA motifs^86^. Regarding W-autosome translocations, despite the unique multiple ♂Z_1_Z_1_Z_2_Z_2_/♀Z_1_Z_2_W sex chromosome system described^1^, recent studies have revealed the fusion of sex chromosomes and autosomes in different bird lineages, indicating that this type of rearrangements is more common than initially believed. For example, the fusion of ZW sex chromosomes with chromosome 11 has been proposed in the ancestor of parrots^21^. Additionally, in the parrot *Myiopsitta monachus*, chromosome 25 has been further fused to the sex chromosomes^21^. In the cuckoo species *Crotophaga ani* a Robertsonian translocation between the microchromosome 17 and the Z chromosome was found^62^. Among songbirds (Sylvoidea), a series of papers have indicated that autosomal material had been integrated into both Z and W^87,88^. Therefore, the evolution of bird W chromosomes is revealing more dynamic than previously thought as new data derived from cutting-edge sequencing and cytogenetic investigations (such as the ones described here) become available.

## Conclusions

Why have birds´ W chromosomes endured for more than 100 Myr? We demonstrate that its evolution could be far more complex than previously thought. We showed that the W chromosomes of both Thrushes did not accumulate higher amounts of heterochromatin and repetitive sequences, as observed in most bird species. Besides, the W chromosome of the pale-breasted Thrush, instead of representing a straightforward "degenerated" version of its earlier homologous Z chromosomes, may represent a dynamic “patchwork” that includes deletions and the integration of new genomic material as a result of chromosomal rearrangements with autosomes. Specific satDNA families were directly associated with these rearranged regions. These findings challenge the unidirectional evolutionary process of W chromosomes widely proposed for birds.

## Methods

### Sampling, chromosomal preparation, and C-banding

The samplings of *T. leucomelas* and *T. rufiventris* were authorized by the Brazilian environmental agency ICMBio/SISBIO (Licenses 61047-4, 44173-1, and 68443-2) and SISGEN (A96FF09). Each individual of *T. leucomelas* and *T. rufiventris* was assigned a code (i.e., male—M; female—F) (Table 2). Mitotic chromosomes were obtained according to the protocols described by Refs.^60,89^, which utilized skin biopsies and bone marrow for fibroblast culture, respectively. The constitutive heterochromatin regions were evidenced following the protocol proposed by Ref.^90^. All experiments followed the guidelines and were approved by the Ethics Committee on Animal Experimentation of the Federal University of Pampa (018/2014 and 019/2020). The authors complied with ARRIVE guidelines.

The genomic DNAs (gDNAs) from *T. leucomelas* and *T. rufiventris* individuals were extracted following the protocol described by Ref.^91^. DNA samples from *T. leucomelas*♀F01 and *T. leucomelas*♂M01 were sequenced using the BGISEQ-500 platform (paired-end 2 × 150 bp) with a 3 × coverage normally required for satellite assembly^25,37^. The genomic reads obtained were deposited in the Sequence Read Archive (SRA) under accession numbers SRR26625300 (male) and SRR26625299 (female).

### Bioinformatic analyses: construction of *T. leucomelas* satellite DNA catalogs and additional analyses

The genomic libraries were subjected to a process of quality trimming using the software Trimmomatic version 0.36 (https://github.com/usadellab/Trimmomatic)^92^. After, the satellitome of a female of *T. leucomelas* was characterized using the TAREAN tool^93^, following the SatMiner pipeline^25^. Then, the outputs containing the putative consensus sequences of satDNAs were used to filter the genomic libraries using the software Deconseq version 0.4.3 (https://deconseq.sourceforge.net)^94^, and other iterations of TAREAN were performed until no satDNAs were found. After the characterization of all consensus sequences, we filtered and removed other tandemly repeated elements, such as multigene families, and a homology search using RepeatMasker^95^ was performed to group the sequences as the same variant (similarity greater than 95%), variants of the same satDNA (similarity between 80 and 95%), and superfamilies (similarity between 50 and 85%), following the patterns established by^25^. The abundance and divergence of each satDNA were estimated in females and males using RepeatMasker software version 3.0 (https://www.repeatmasker.org)^95^, with a random selection of 2 × 5,000,000 reads. After that, satDNA families were named according to their abundance in *T. leucomelas*. Considering the particularities of the sex chromosome system of *T. leucomelas*, the quotient between the abundance of each satDNA in females and males (F/M) was calculated to verify putatively accumulated satDNAs in the sex chromosomes. TleSatDNAs were deposited in GenBank with accession numbers OR675141.1–OR675150.

Besides, we selected TleSat05-21 and TleSat07-103 to construct minimum spanning trees (MSTs). Only these satDNAs were selected due to technological limitations, in which it is only possible to use satDNAs whose monomer size is smaller than the read size (< 150 bp in this case). We extracted monomers of the cited satDNAs from genomic libraries of both sexes, followed by alignment of the reads against each satDNA, to select only full reads. After that, we discarded singletons using CD-Hit software version V4.8.1 (https://sites.google.com/view/cd-hit/home?authuser=0)^96^. Finally, the MSTs were constructed using PHILOVIZ 2.0 software version 2.0 (https://www.phyloviz.net)^97^, and Inkscape was utilized to produce the final image.

### Primer design and amplification using polymerase chain reaction (PCR)

A total of ten satDNA sequences (hereafter named TleSatDNAs) were isolated (Table 1), for which eight were designed primers (TleSat01, TleSat02, TleSat03, TleSat06, TleSat07, TleSat08, TleSat09, and TleSat10). The remaining two (TleSat04 and TleSat05) were synthesized and labeled with Cy3 at the 5' end by ThermoFisher (ThermoFisher Scientific), since they are smaller than 30 bp. The PCR reactions followed the conditions optimized according to^35^. To confirm the amplification of each satDNA, the PCR products were subjected to electrophoresis in a 1% or 2% agarose gel, and subsequently quantified by the ThermoFisher NanoDrop spectrophotometer (ThermoFisher Scientific).

### Fluorescence in situ hybridization (FISH)

All TleSatDNAs were labeled using a nick translation Kit from Jena Bioscience (Jena, Germany) incorporating the fluorophore Atto488-dUTP or Atto550-dUTP according to the instructions in the manufacturer's manual. Microsatellite sequences (GAA)_10_, (GAC)_10_, (CGG)_10_, (CAC)_10_, (CAG)_10_, (CAT)_10_, (GAG)_10_, (TAA)_10_, (TAC)_10_, (CAA)_10,_ (GA)_15_, (CA)_15_, (GC)_15_, (TA)_15_, (C)_30_, and (A)_30_ were labeled directly with Cy3 at the 5' end during synthesis (VBC Biotech, Vienna, Austria) and also used in the hybridization experiments. We performed the fluorescence in situ hybridization experiments following the protocol described by^98^. The slides were dehydrated in a 70%, 85%, and 100% ethanol solution and the metaphases were stained with 4',6-diamidino-2-phenylindole (DAPI).

### Comparative genomic hybridization (CGH)

We performed intraspecific CGH in both *T. leucomelas* and *T. rufiventris* individuals following the experimental designs described in Table 3. For this purpose, gDNAs from males and females of each species were respectively labeled using a nick-translation labeling kit with Atto488-dUTP (green) and Atto550-dUTP (red), from Jena Bioscience (Jena, Germany). To block common genomic repetitive regions, we used Cot-1 DNA derived from the male gDNA of each species, produced according to^99^. Each hybridization was composed of 3 µg of male-derived Cot-1 DNA and 500 ng of each labeled male and female gDNAs. After using ethanol-precipitation, the pellet was air-dried and well mixed with 20μL of hybridization buffer (Denhardt's buffer, pH 7.0), composed of 50% formamide, 2% 2xSSC, 10% SDS, 10% dextran sulfate. The CGH experiments followed the methodology detailed in^100^. After the CGH experiments, the *T. leucomelas* chromosomal slides were washed 3 times in a 4SSC-Tween solution at 42 °C, and sequentially in situ mapped with TleSat06 (red) and TleSat08 (green) probes. Finally, the material was sequentially C-banded using the abovementioned probes and protocols.

**Table 3:** The experimental design used for intraspecific comparative genomic hybridization indicating the chromosomal background, the gDNAs probes applied, and the corresponding results.

Chromosomal background	gDNA probes	Results
TRU ♀F01	♀F01 × ♂M01	Figure 5
TRU ♀F02	♀F02 × ♂M01	Data not shown
TRU ♂M01	♀F01 × ♂M01	Suppl. Fig. S2E
TRU ♂M02	♀F01 × ♂M02	Data not shown
TLE ♂M01	♀F01 × ♂M01	Figure 6A and Suppl. Fig. S2A
TLE ♂M02	♀F01 × ♂M02	Figure 6B and Suppl. Fig. S2B
TLE ♂M03	♀F01 × ♂M03	Figure 6C and Suppl. Fig. S2C
TLE ♂M04	♀F01 × ♂M04	Suppl. Fig. S2D
TLE ♀F01	♀F01 × ♂M01	Figure 6D and Suppl. Fig. S3A
TLE ♀F02	♀F02 × ♂M01	Suppl. Fig. S3B

### Microscopic analysis and image processing

To corroborate the 2n, karyotype structure, FISH, and CGH results, at least 30 metaphase spreads per individual were examined. Images were captured using an Olympus BX50 microscope (Olympus Corporation, Ishikawa, Japan), with CoolSNAP, and the images were processed using Image-Pro Plus software version 4.1 (https://mediacy.com/image-pro) (Media Cybernetics, Silver Spring, MD, USA). Chromosomes were classified according to^101^.

## Supplementary Information

Supplementary Figures.

## Data Availability

The datasets generated during and/or analyzed during the current study are available from the corresponding author on reasonable request. The catalog of satellite DNAs was deposited on the GenBank with accession numbers OR675141.1- OR675150 and raw reads are available in Sequence Read Archive (SRA-NCBI) under accession numbers SRR26625300 (male) and SRR26625299 (female).
