# Supplementary material for: Satellitome analysis on the pale-breasted thrush Turdus leucomelas (Passeriformes; Turdidae) uncovers the putative co-evolution of sex chromosomes and satellite DNAs
Source: Sci Rep. 2024 Sep 4;14:20656. doi: 10.1038/s41598-024-71635-5 (PMC11375038; doi:10.1038/s41598-024-71635-5)
Supplement: Supplementary file 1 — Supplementary Figures. [file 41598_2024_71635_MOESM1_ESM.docx]

**Supplementary Figures**

**
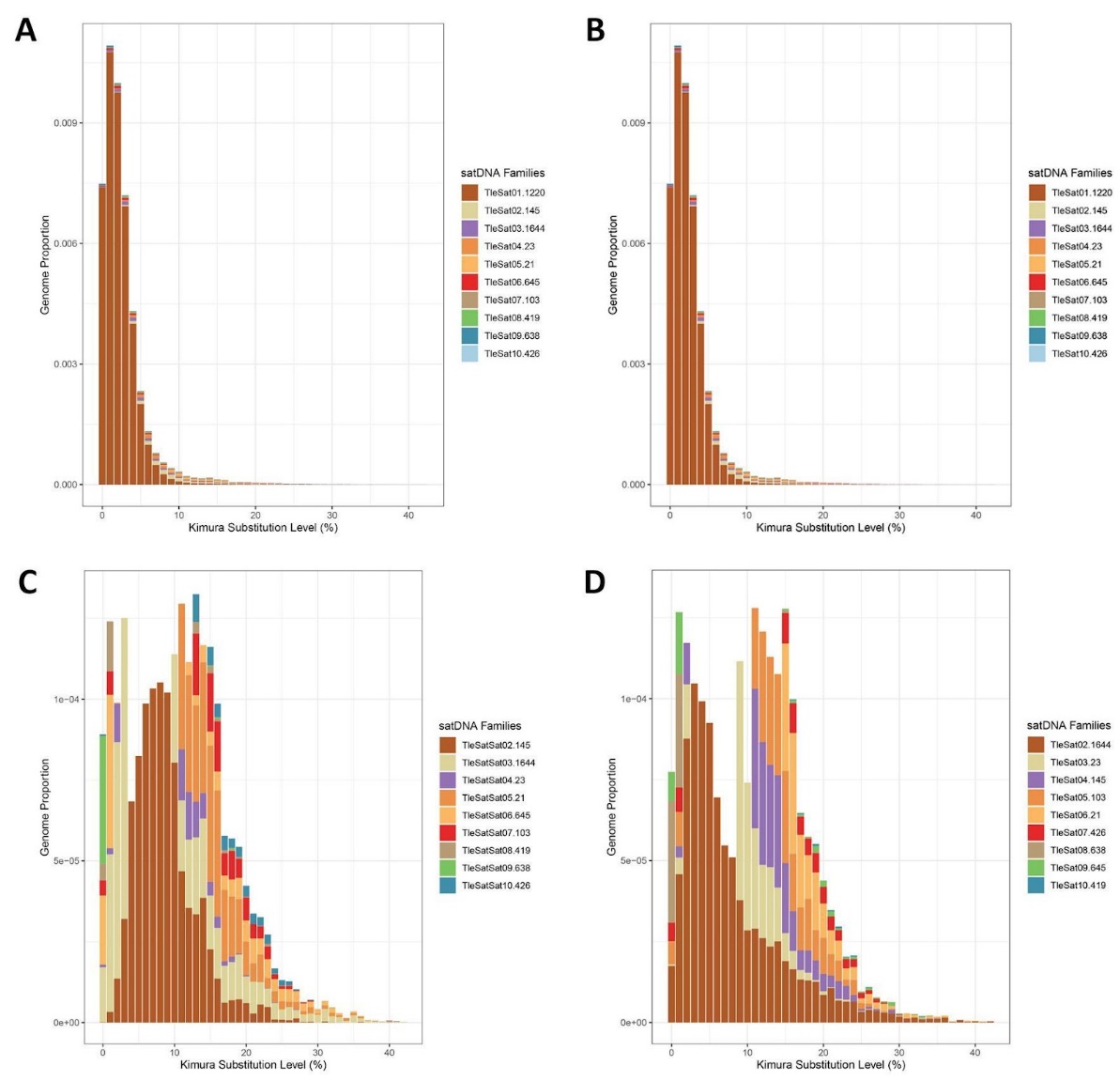
**

**Supplementary Fig. 1.** Repeat landscapes of the TleSatDNAs in female (A) and male (B) of TLE. In addition, repeat landscapes of females (C) and males (D) suppress TleSat01–1220.


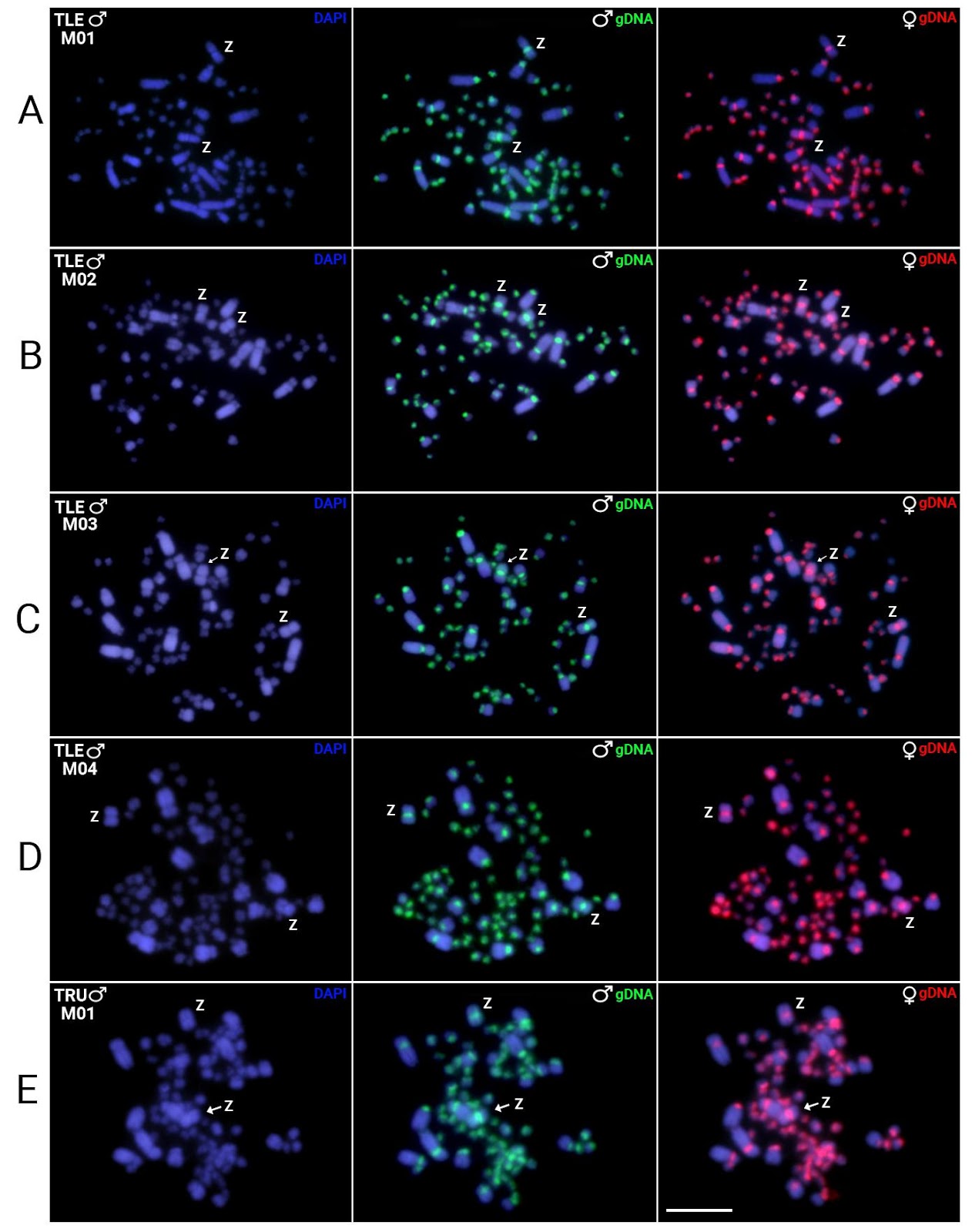


**Supplementary Fig. 2** Comparative genomic hybridization in all the four *T. leucomelas* and one *T. rufiventris* male individuals analyzed in the present work: TLE♂M01, TLE♂M03, TLE♂M04 (A, C, D) from Porto Vera Cruz (RS), TLE♂M02 from Belém (PA) (B), and TRU♂M01 (E). The first column corresponds to DAPI, the second to male gDNA, and the last one to female gDNA. The marged images are located in Figure 6 and 7. Both TLE♂M03 and TLE♂M04 showed similar patterns of intragenomic-CGH, for this reason only TLE♂M03 is represent on Fig. 6.


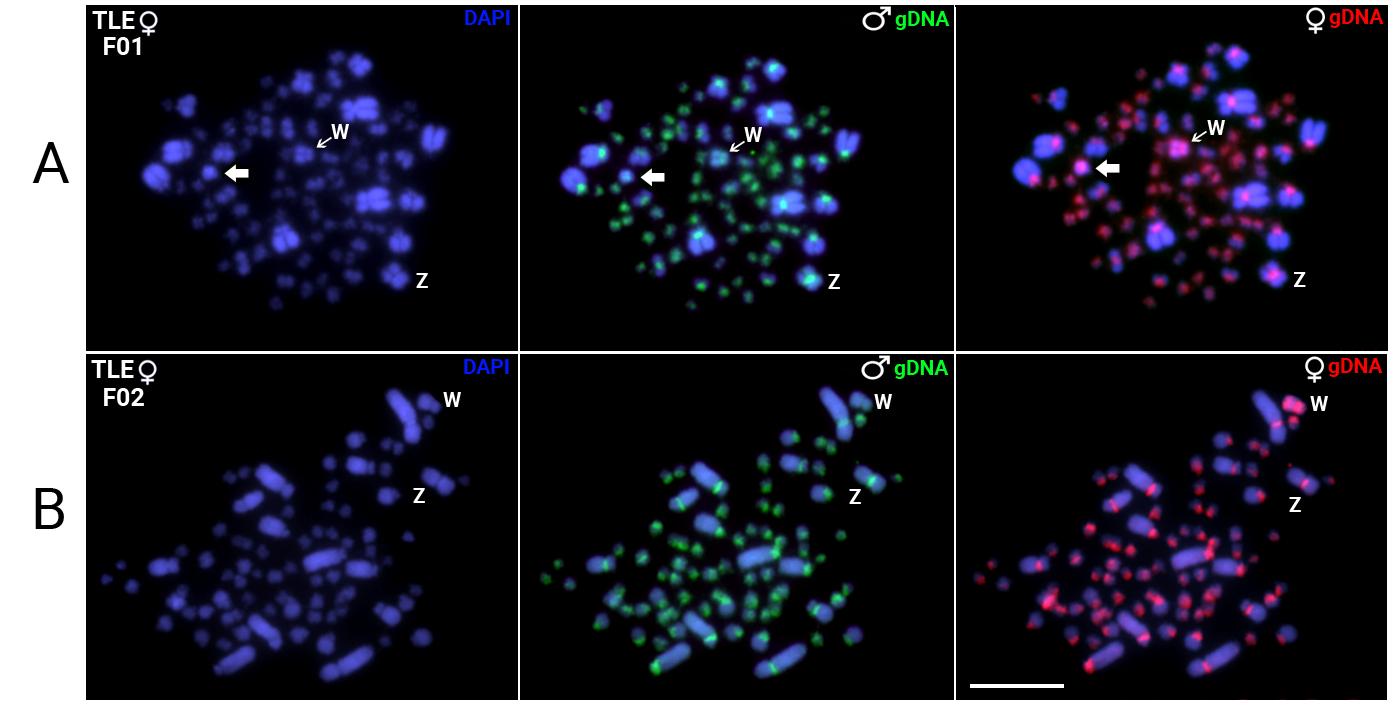


**Supplementary Fig. 3** Comparative genomic hybridization in all the two *T. leucomelas* female individuals analyzed in the present work: TLE♀F01 and TLE♀F02. The first column corresponds to DAPI, the second to male gDNA, and the last one to female gDNA. The merged images are located in Fig. 7. Both TLE♀F01 and TLE♀F02 showed similar patterns of intragenomic-CGH, for this reason only TLE♀F01 is represented in Fig. 6.
